# Cluster-Based Relocation of Stations for Efficient Forest Fire Management in the Province of Valencia (Spain)

**DOI:** 10.3390/s21030797

**Published:** 2021-01-25

**Authors:** Miguel de Domingo, Nuria Ortigosa, Javier Sevilla, Sandra Roger

**Affiliations:** 1 Computer Science Department, Universitat de València, Av. de la Universitat s/n, 46100 Burjassot, Spain; nuorar@upvnet.upv.es (N.O.); javier.sevilla@uv.es (J.S.); 2 I.U. Matemática Pura y Aplicada, Universitat Politècnica de València, Camino de Vera s/n, 46022 València, Spain

**Keywords:** fire prevention, artificial intelligence, k-means, DBSCAN, Floyd–Warshall

## Abstract

Forest fires are undesirable situations with tremendous impacts on wildlife and people’s lives. Reaching them quickly is essential to slowing down their expansion and putting them out in an effective manner. This work proposes an optimized distribution of fire stations in the province of Valencia (Spain) to minimize the impacts of forest fires. Using historical data about fires in the Valencia province, together with the location information about existing fire stations and municipalities, two different clustering techniques have been applied. Floyd–Warshall dynamic programming algorithm has been used to estimate the average times to reach fires among municipalities and fire stations in order to quantify the impacts of station relocation. The minimization was done approximately through *k*-means clustering. The outcomes with different numbers of clusters determined a predicted tradeoff between reducing the time and the cost of more stations. The results show that the proposed relocation of fire stations generally ensures faster arrival to the municipalities compared to the current disposition of fire stations. In addition, deployment costs associated with station relocation are also of paramount importance, so this factor was also taken into account in the proposed approach.

## 1. Introduction

Fires are a tremendous threat to both urban areas and natural ecosystems, often leading to chaotic, even critical situations. Since forest fires represent a problem for climate change, it is worrying that these natural disasters have increased in recent decades. Early fire warning systems allow one to fight against these natural disasters. They are mostly based on camera surveillance applications in home monitoring [1,2], or wireless sensor networks for large green areas [3,4]. Exhaustive surveys on different technologies used for forest fire detection have been carried out during the last few years [5]. Wireless sensor networks [6] and mobile ad hoc networks [7] are two significant techniques in the context of firefighting, whereas drone swarms have also been recently proposed for both fire detection and fire extinguishing [8]. In addition, the strengths and weaknesses of fire detection systems based on optical remote sensing are deeply analyzed in [9], those including sensors placed on the ground, in the air and in outer space.

However, prevention is better than a cure, so fire prediction techniques may help to identify which areas that are at higher risk of fires. For example, Guldaker [10] found and predicted risk areas for residential fires based on geo-visualization techniques. Regarding forest fire prediction, parameters such as previous weather conditions, humidity and cumulative precipitation can offer a risk estimate by means of artificial intelligence methods, such as support vector machines and artificial neural networks [11,12], and fire ignitions caused by negligence and pyromaniacal behavior can also provide valuable information in the prediction [13].

Above-mentioned factors, along with population density and accessibility limitations, are commonly included when defining prevention planning and emergency management for forest fires [14]. Orography complexity has also been analyzed to develop projective geo-referencing algorithms for decision support aids for fire risk evaluation and monitoring [15].

In general, the placement of fire stations plays a major role on the effectiveness provided by the fire department [16]. Localization requirements are different depending on the scenario [17]. Travel times from fire stations are always critical, but even more so when the fire is located in the surroundings of a petrol station [18] or in high-density population areas [19,20].

Several previous works made use of geographic information systems (GIS) for aiding fire service optimization [21]. GIS has also been used to calculate the optimized route based on distance and time of travel, slope of the roads and delays [22,23], and to minimize the total cost by mathematical models which include proper features of the area under analysis as the location in earthquake zones [24]. Related to this, fire simulations with forest harvest scheduling techniques have also been proposed to model schedule management activities and optimize them to mitigate wildfire damage [25]. When optimizing management activities, not only are heuristic methods used, but operational and fuel consumption reasons are considered too.

The determination and optimization of fire stations’ locations is a crucial task to improving the emergency coverage. Different approaches have been studied, such as constraint-based solutions to generate the Pareto frontier to explore alternative fire station location scenarios [26]; hierarchical covering models to deploy macro and micro fire stations according to different capacities [27]; mixed-integer linear programming models which consider multiple regions and vehicles average utilization to calculate demand types [28]; and multiple criteria decision analysis to be applied after mergers of different fire brigades [29].

Fires in the Mediterranean basin include a significant percentage of the total fires occurring worldwide [14,30,31]. In particular, during the last decade, the province of Valencia in Spain has suffered some extremely serious fires. So much so that, in 2012, Valencia province suffered two devastating fires: one in the municipality of Cortes de Pallás and the other one in Andilla. The first one is considered the most catastrophic fire of the 21st century in Spain, which burned more than 30,000 hectares. Even so, Andilla’s fire was not less important, because it is considered the third most destructive fire of the century [32]. In total, adding both, it is estimated that about 20 municipalities were affected [33]. Although there was already some work on fire prevention done by the Valencian Government, also known as *Generalitat Valenciana* (GVA), such as their integrated system for forest fire management (SIGIF) [34], these fires might have increased the awareness level in this field.

In this context, the aim of this paper is to propose a new redistribution of forest fire stations in the province of Valencia in order to optimize their locations by means of artificial intelligence techniques. An intelligent relocation of stations could maximize the protection of villages while reducing the wildfires’ impact in the environment. The proposed approach is aimed at avoiding the fast spread of forest fires through reducing the time it takes to reach them. The time to reach a fire may be straightforwardly reduced by increasing the number of stations, but this would also imply high deployments costs. This paper aims to find a balance between shortening the arrival time and avoiding high investment costs due to station redistribution. To this end, the minimization of arrival time was done approximately by a clustering algorithm. The outcomes of applying k-means clustering with different numbers of clusters determined a predicted tradeoff between reducing the time and the cost of more stations.

## 2. Materials and Methods

This section presents first how the information about the municipalities in the province of Valencia is represented to enable its processing in the proposed approach. We refer to this step as land mapping. After that, the representation of fire data is described by means of a matrix. Then, the bases for the two clustering methods used in the proposed approach are detailed, and the algorithm to calculate the distances between the different municipalities and from them to their nearest fire station is described.

### 2.1. Land Mapping

**Figure 1:**
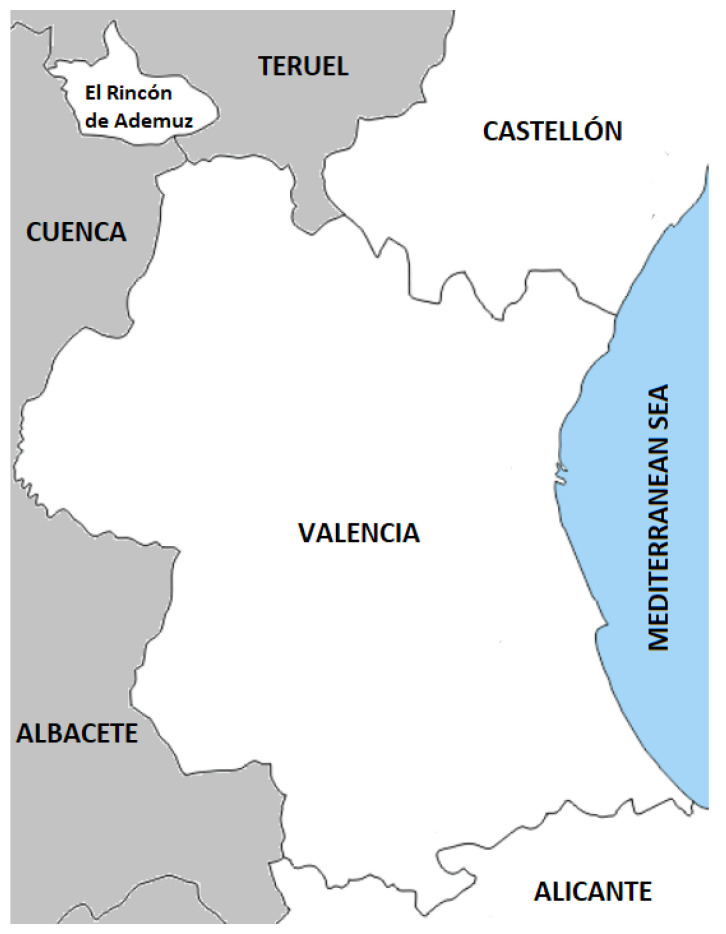
Shape of the province of Valencia.

Figure 1 represents the province of Valencia and its surrounding provinces. The first step is to represent the region of interest in a way that is useful for carrying out the proposed optimization of station positions. Considering the extension of the land, which is 10,763 km${}^{2}$, it was decided to select 266 points within the province, one for each municipality in the province. These points were selected according to two conditions: (1) they had to belong to the urban area of their corresponding municipality and (2) they had to lie on a main street or road. From a set of points that met these criteria for the same municipality, a representative point was chosen randomly.

Once the points were selected, a directed graph was used, wherein the province’s municipalities and corresponding points are its vertices, and their edges are the distances between them. At the same time, an identifier ranging from 0 to 255 was assigned to each municipality, so that a 266×266 matrix M representing the graph distances could be established. In this way, to find out how long it takes to go from point 0 to point 1, the position (0,1) of the matrix M must be looked at.

**Figure 2:**
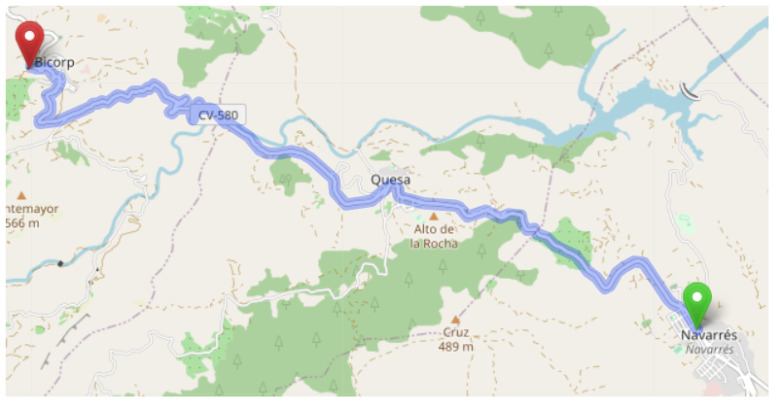
Shortest path between Navarrés and Bicorp.

The next step was to obtain the distance between each pair of municipalities (i.e., the entries of M). To this end, widely used online map applications such as Google Maps are very useful, where time and distance are easily obtained by defining the starting and finishing points. After the application returns time and distance of paths, only the fastest given path is selected. In case two paths have the same duration, the shortest one is selected. However, note that if that mechanism is followed, there would be more than 70,000 application requests to be done (more specifically, 265 for each municipality), which would result in a very cumbersome task. For this reason, instead of complete routes between municipalities, point-to-point distances was used. Here is an example to illustrate how point-to-point measurements are done. Figure 2 represents in a realistic map the fastest route between the two villages of Navarrés and Bicorp. It can be observed that, since the route passes through the urban area of another village (Quesa), the time to go directly from Navarrés to Bicorp was be requested; rather, it was calculated as the sum of the time to go from Navarrés to Quesa and the time to go from Quesa to Bicorp. Following this reasoning, algorithms aimed at finding the shortest paths between all the points on a map could be used, in order to provide accurate results.

The following step was aimed at preparing the data for the subsequent execution of the clustering method. The province was then represented by means of a new matrix P, which, in this case, allowed us to map each municipality to a matrix position according to its actual location. As shown by Figure 1, the province of Valencia does not have a regular (e.g., square or rectangular) shape, which would naturally fit within a matrix structure, even less since the region named *El Rincón de Ademuz* also belongs to it. For this reason, it is assumed that the matrix P will be not only composed of cells from the whole area of the province, but also of certain regions of the adjacent provinces, taking into account the big sea area covered by it as well.

To establish the boundaries of the matrix, four villages were considered: the most north-lying (Castielfabib), the most south-lying (Bocairent), the most east-lying (Oliva) and the most west-lying (Villargordo del Cabriel). After that, both the width and the height of the matrix had to be determined. On the one hand, the distance along the north–south axis was measured as the distance in a straight line between the latitude of Castielfabib and the latitude of Bocairent, fixing the same longitude for both coordinates. On the other hand, the same system was used for the east–west axis, but just exchanging latitude by longitude and considering Oliva and Villargordo del Cabriel. The territory was into square areas or cells, which were mapped to the entries of a matrix P, considering a correspondence between the east–west axis and the matrix columns, and between the north–south axis and the rows. As a result, the top left and bottom right corners of the map are represented, respectively, by the (0,0) and (255,255) matrix positions.

**Figure 3:**
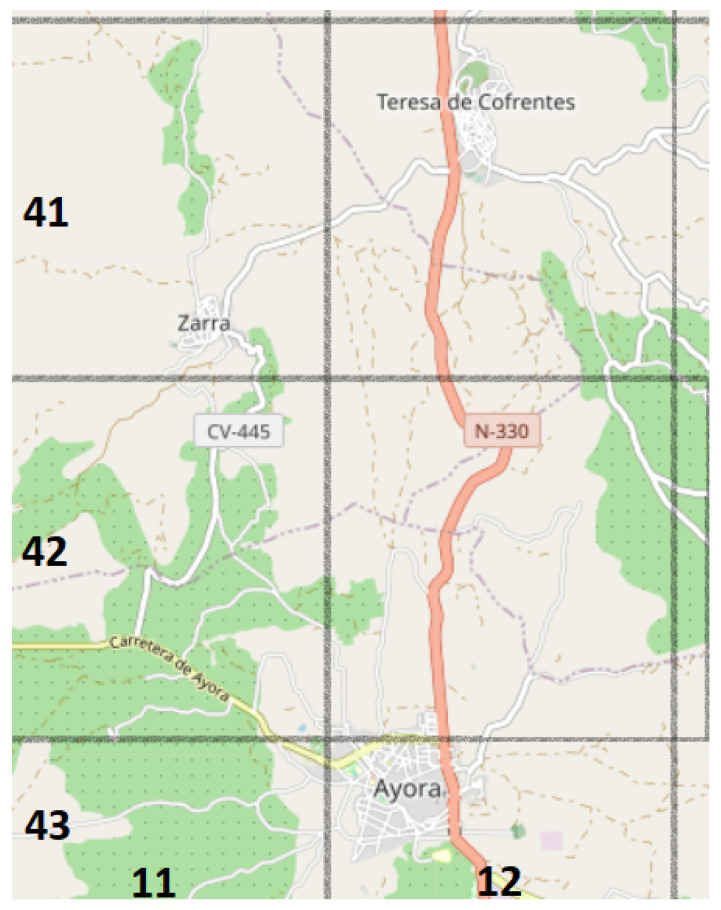
Small part of the province divided as the matrix P would do. The numbers at the bottom indicate the column numbers and those on the right the row numbers. Painted in red, there is the road linking two villages: Ayora and Teresa de Cofrentes.

It was observed that the target region is around 150 km long and 117 km wide. To avoid the usage of large matrices, square areas of quasi 7.5 km${}^{2}$ were defined, resulting in a matrix P of size 55×43. The cell size was selected because of the following reasons. Assuming a straight line running through the cell horizontally or vertically, the line would be approximately 2.7 km long. Taking this into account, and for an average road speed of 100 km/h, the line could be traveled from end to end in less than two minutes. Obviously, the roads are not completely straight in reality, so it could be assumed that it takes about two minutes to go through a cell, as is shown by Figure 3. In reality, the route between Ayora and Teresa de Cofrentes takes about four minutes. According to the assumptions made, a two-cell journey should cost the same. That is why this cell size is considered to be a suitable way to map the territory, since it has a certain extension, but it can be crossed quickly under normal circumstances.

In this way, each municipality was assigned to the matrix entry in P according to its corresponding point’s location. It can happen that, in some cases, the point is right on the border between two or four square areas. In that case, the matrix position must be assigned to guarantee that most of the urban area of the municipality is located in that square area. Applying this method, every municipality would have its corresponding coordinates with a certain reliability.

### 2.2. Management of Fire Data

Although the GVA has a larger fire archive, only the fires that occurred between the 1st of January 2000 and the 31st of December 2015 have been considered in this work. Data have only been chosen in this time interval, because the information from some regions was missing when going back in time, and from 2016 on, there were still some fire reports that were incomplete.

To organize the necessary information, each fire was assigned to the municipality in which it started. The matrix P was used to organize the number of fires in each municipality too. Thus, if, for instance, 15 fires began in the village linked to the matrix P position (10,10), the matrix entry would have the value 15, representing the fires that began at the mentioned position. To adapt this problem to the algorithms, the fires in each municipality would act as data points and each fire station would correspond to a cluster. Therefore, varying the number of clusters would change the number of fire stations in the same way.

### 2.3. Data Clustering Algorithms

Clustering is a widely known type of unsupervised learning technique in the field of machine learning [35]. It may be useful when there is no labeled data. Several clustering algorithms are found in the literature [36]. Next, we detail two of these algorithms which have been used in the proposed approach of this paper: a partition-based clustering algorithm which needs the initial number of clusters (*k*-means),and a density-based one (DBSCAN).

#### 2.3.1. Partition-Based Clustering: *k*-Means

The *k*-means clustering algorithm is one of the many existing algorithms where the clustering process consists of partitioning a set of data points into subsets of clusters, so that points within the same cluster are similar to each other, while those are not that similar belong to other clusters [37]. *k*-means has been widely used in multimedia programming, computer vision and machine learning for vector grouping and quantification [38].

The standard *k*-means method, also known as Lloyd’s algorithm, is an iterative refinement method that effectively minimizes the distance between each point and its assigned cluster center (centroid) [39]. This method requires as input parameters the number of clusters (*k*) and the distance metric that will be used (in our case, the squared Euclidean distance).

The set of *n* samples is denoted as *X*, which are assigned to *k* disjoint clusters *C*. The *k*-means algorithm is first initialized with *k* random seeds to choose the initial centroids. Then, two steps are iteratively performed: assignment and refresh. The assignment step finds the nearest cluster for each point by checking the distance between that point and each centroid. The refresh step recalculates the cluster centers based on the average of all data points assigned to each centroid. Hence, *k*-means aims to calculate the centroids such that variance is minimized (i.e., the within-cluster sum of squares is minimized):

(1) $$\sum_{j=0}^{n}\underset{\mu_{i}\in C}{min}\left(|\right|x_{j}-\mu_{j}{\left|\right|}^{2})$$

where $\mu_{j}$ is the mean of the samples in cluster (centroid of cluster $C_{j}$). Thus, the distance between each point and the centroid is obtained. Centroids are iteratively recalculated and all data points are relabeled until no significant changes in the centroids location are observed at the new step.

#### 2.3.2. Density-Based Spatial Clustering: DBSCAN

Density-based spatial clustering of applications with noise (DBSCAN) is another well-known clustering algorithm commonly used in machine learning [40]. In contrast to *k*-means, this technique does not initially require one to set the number of clusters (*k*), since DBSCAN groups together points close enough to be considered a cluster, if there is a minimum number to form one.

DBSCAN requires two parameters to define the density: the maximum distance between two data points to be considered as neighbors (ϵ), and the minimum number of neighbors to form a core sample (*minPoints*). Core samples are described as areas of high density, whereas a cluster is defined as a set of core samples which are close to each other.

Thus, DBSCAN algorithm considers as different clusters the diverse high-density areas separated by low-density areas. The algorithm starts with a point *x* of the data set *X*, and obtains all neighbors of *x* which are located at a distance lower than ϵ. If the number of neighbors is greater the number of *minPoints*, then *x* is considered a core sample and a new cluster is created, formed by *x* and its neighbors. Then, the algorithm iteratively collects the neighbors within ϵ distance from the different core samples. The process finishes when all the data points have been processed.

DBSCAN parameters can be estimated by several techniques, such as k-distance graph or an OPTICS (ordering points to identify the clustering structure) plot [41]. According to them, DBSCAN marks as outliers the points that are in low-density regions. This algorithm facilitates clusters found by DBSCAN to be of any arbitrary shape, as opposed to *k*-means, which assumes that clusters are convex-shaped.

### 2.4. Floyd–Warshall Algorithm

In this section, the focus is on presenting an efficient way to estimate the time to reach all the municipalities from their nearest fire stations. This calculation was necessary and very useful for evaluating the performance of the proposed fire station relocation.

A similar problem often arises when making a table of distances between all pairs of cities to establish a road map [42]. This is done by adapting the map as a directed and weighted graph G=(V,E) with a cost function w=E→R, which represents the real-valued distance of each edge. For each pair of vertices u,v∈V, the goal is to find the fastest route, where the value of the route is the sum of all costs of the edges that compose it. The output of this problem is often presented as a table where the entry in row *u* and column *v* should be the shortest path between these two edges. Well-known methods for shortest-path calculations are Dijkstra’s and Bellman–Ford algorithms [42], which are classified as single-source because they require multiple runs (*V*), one for each vertex acting as a source. Single-source algorithms represent the graph by means of an adjacency list.

In this work we make use of the Floyd–Warshall algorithm, which is more efficient than the aforementioned single-source algorithms. In this algorithm, the graph features are represented through an adjacency matrix instead of an adjacency list. In particular, it is assumed that each vertex is numbered within the range [1,V], so that a matrix W with size V×V represents the edge weights of a directed graph G=(V,E). In that case, the weights for each i,j∈V fulfill that:

(2) $$W_{ij}=\left\{\begin{array}{cc} 0, & \mathrm{if}i=j,\\ \mathrm{weight}\mathrm{of}(i,j), & \mathrm{if}i\neq j\mathrm{and}(i,j)\in E,\\ \infty , & \mathrm{if}i\neq j\mathrm{and}(i,j)\notin E, \end{array}\right.$$

where a zero value is used to represent the connection of one vertex with itself, whereas an infinity value is used where there is no connection among the two vertices.

The Floyd–Warshall algorithm bases its assumption on the fact that there is a subset of intermediate vertices κ={1,2,...,k} for any pair of vertices i,j∈V [42]. According to this statement, all paths from *i* to *j* whose intermediate vertices are in the subset κ must be considered. Thus, following the same scheme as in the previous equation, and being *x* any vertex in the subset κ, the matrix elements are obtained as follows:

(3) $$W_{ij}=\left\{\begin{array}{cc} W_{ij}, & \mathrm{if}W_{ij}\leq W_{ix}+W_{xj},\\ W_{ix}+W_{xj}, & \mathrm{if}W_{ij}\geq W_{ix}+W_{xj}. \end{array}\right.$$

Note that after executing the previous instruction with all possible combinations of the three variables i,j and *k* within the range [1,V], the shortest path for all possible cases would be found.

As it will be elaborated in the next section, the Floyd–Warshall algorithm will be applied over the matrix representing the graph distances for all the municipalities.

## 3. Proposed Approach

With the aim of minimizing the impact of forest fires in Valencia province, an optimized distribution of forest fire stations is proposed, which is able to reduce the average travel time between fire stations and the different municipalities. The proposal considers the following relevant input information:

- Location information about existing forest fire stations. This information was obtained from public records published by the Valencian Agency for Safety and Emergency Response.
- Historical data about forest fires in the Valencia province. This information was also obtained from public records, more specifically from the integrated forest fire management system developed by the fire prevention service of the GVA.
- Distances between all pairs of adjacent municipalities measured in travel time. This information was collected from publicly available online map applications. In particular, Google Maps has been used.

Once the relevant input information was collected, we proceeded to apply the clustering methods to obtain the new fire station locations. Then, the shortest path to reach each one of the different municipalities from these optimized locations by means of the Floyd–Warshall algorithm was calculated. A detailed description of these two processes is included in the next two subsections.

### 3.1. Fire Station Relocation

In order to get optimized locations of the forest fire stations, two different clustering algorithms were evaluated. In this case, results for *k*-means and DBSCAN have been obtained. To this end, the corresponding packages of the standard *Scikit-learn* library in Python [43] have been used in the proposed implementation.

**Figure 4:**
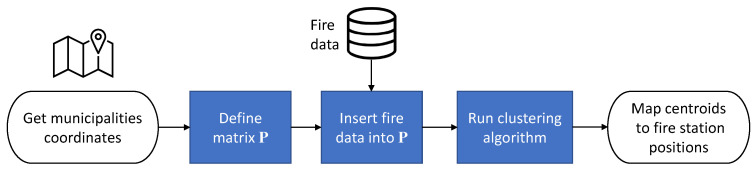
Block diagram of the proposed approach for fire station relocation.

Figure 4 shows the block diagram of the fire station relocation proposal. In a first step, matrix P is obtained using the historical fire data. Then, the new fire station placement is calculated through either the *k*-means or the DBSCAN clustering technique.

Both clustering methods provide consistent solutions, in which the obtained centroids or core samples correspond to the optimized fire station positions. However, the procedure to get the results is different from one algorithm to another.

In the *k*-means algorithm, the number of clusters is chosen at the beginning of the execution. However, the solutions may differ in distinct executions with the same number of clusters, because the centroids are initialized randomly. For this reason, it was decided to run each algorithm 1000 times and extract the results based on the most repeated matrix positions.

Concerning the execution of the DBSCAN method, its peculiarity is that, instead of the desired number of clusters, it requires as input parameters the maximum distances between data points to be considered as neighbors (ϵ), and the minimum number of data points needed to define an independent cluster (*minPoints*). Then, the algorithm returns the optimum number of clusters together with their core samples. To optimize the selection of the input parameters, an exhaustive experimentation was carried out to maximize the F1-score [44] while minimizing the distance between the different data points and the cluster center. In particular, ϵ was given values ranging between 1.0 (the minimal distance between two fires considering the cells’ distribution) and 21.6 (the average distance), whereas *minPoints* took values varying from 2 to 17 (the minimal number of fires per point).

Thus, as previously indicated, fire stations are initially located on each one of the obtained centroids or core samples. Even so, there is one main factor to be considered once the definitive positions of all centroids are obtained—that is, the road. There are two possibilities: either the centroid position is accessible by road or it is not. For instance, if a centroid lies within a green or poorly communicated area, this station will have to be relocated to the closest possible area that is accessible by road, despite its original position being the optimum one according to the algorithm criteria. Otherwise, the time costs for accessing the municipalities would significantly increase.

Once the road points where the fire stations must be located near by have been fixed, two new possibilities appear. On the one hand, the chosen point could be within the urban area of a municipality. In those cases, the time to reach the respective municipality is set to 0. On the other hand, if the selected point is not in any urban area, the time to reach the adjacent municipalities needs to be obtained. It is then when the Floyd–Warshall algorithm must be executed to obtain the definitive distances among all the selected points in the map, and thus, to allow measuring the time it takes to reach each municipality. This is detailed in the next subsection.

### 3.2. Shortest Path Calculation

**Figure 5:**
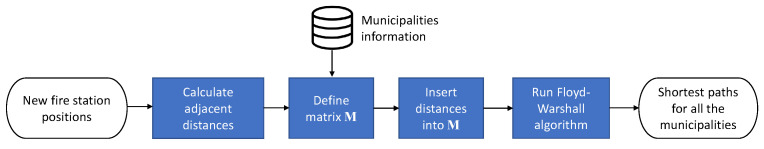
Block diagram of the proposed approach for shortest path calculation.

Once the new fire stations locations were determined, the distances between them and their adjacent villages were collected by using Google Maps. In addition to providing route times, Google Maps considers the road sections that are usually congested, and in those cases, shows a slower route time than it would be if the traffic were fluid. These point-to-point distances were stored in matrix M, which was introduced in Section 2.1 as the matrix representing the graph distances for all the municipalities. After this, the distances between pairs of non-adjacent villages or fire stations were obtained by means of the Floyd–Warshall dynamic programming algorithm for shortest-path calculation. This is depicted in the block diagram shown in Figure 5.

## 4. Results

This section presents the obtained results in terms of time to reach a fire and the optimized locations of forest fire stations according to the proposed approach. In a first step, results are presented with full freedom to redistribute all fire stations. After that, we discuss the results and propose an alternative constrained approach with limited redistribution of fire stations, while setting some restrictions to reduce the economic impact of the whole fire station redistribution.

### 4.1. Time to Reach a Fire

The calculation of the average time $t_{avg}$ to reach a fire consists of adding up the time of arrival to each village $t_{n}$ and dividing it by the number of municipalities $N_{m}$.

(4) $$t_{avg}=\frac{1}{N_{m}}\sum_{n=1}^{N_{m}}t_{n}.$$

The calculation of the weighted time $t_{w}$ considers the probability of a fire occurring in each municipality ($p_{n}$). These probabilities are approximated as relative frequencies, i.e., calculated as $p_{n}=f_{n}/N_{f}$, where $f_{n}$ is the number of fires produced in the *n*-th municipality and $N_{f}$ is the total number of fires in the province between 2000 and 2015. The resulting weighted time is calculated as:

(5) $$t_{w}=\sum_{n=1}^{N_{m}}p_{n}t_{n}$$

Given that the province of Valencia currently has 26 forest fire stations, we have studied different distributions by varying the number of stations from 20 to 30, aiming at finding a number of fire stations that provides the shortest time to reach each fire compared to the rest. Table 1 represents both the average and weighted times to reach a municipality after running *k*-means and DBSCAN for different numbers of clusters.

It can be observed in Table 1 that the results provided by *k*-means present faster times than the current fire station distribution with 26 stations, whose average and weighted times are 12${}^{\prime }$37${}^{\prime }$${}^{\prime }$ and 10${}^{\prime }$05${}^{\prime }$${}^{\prime }$, respectively. This occurs even if the number of fire stations is reduced to 20. Furthermore, if we compare the DBSCAN results with the ones obtained using *k*-means, this last one undoubtedly provides a better fire stations redistribution.

From the results, it can be also seen that the density-based logic of DBSCAN does not provide an adequate solution to the proposed problem, since the algorithm provides slower times than the current distribution. In this case, the distribution of the data is not the most appropriate to apply a density-based clustering algorithm. On the one hand, the province of Valencia contains some areas where the density of villages is very high. These areas are characterized by a low to medium number of fires per village, which implies that in the corresponding entries for the matrix P, practically every cell is associated with one or more villages. Therefore, almost all cells could contain fire data points, although the fire incidence is not too high. On the other hand, there are other areas where villages are more separated from each other and also tend to have a higher number of fires. For this reason, the cells corresponding to the most separated villages have high densities of points. This fact can, in some way, mislead the algorithm’s logic, causing inaccurate results. Thus, in the remainder of the manuscript we will focus only on results provided by the *k*-means clustering method.

**Figure 6:**
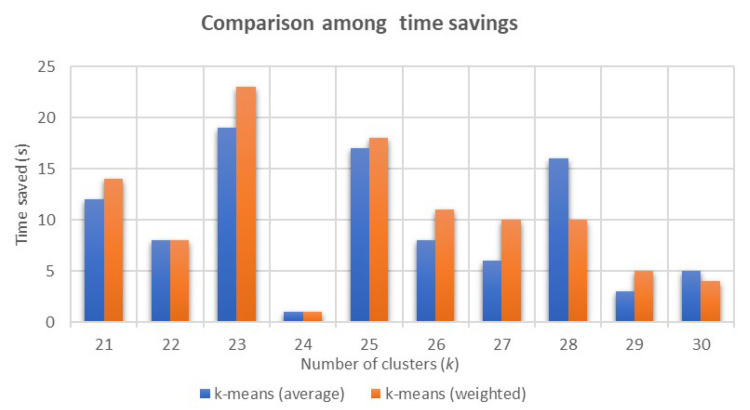
Evolution of time saved with respect to having one cluster less for different numbers of clusters.

As the number of clusters increases, the different *k*-means distributions produce faster times to reach fires with a clear trend. While in the interval [20,25] there is a gain of 57${}^{\prime }$${}^{\prime }$ in terms of $t_{avg}$, in the interval [25,30] this gain is reduced to 38${}^{\prime }$${}^{\prime }$. In order to display this data in a more visual way, Figure 6 represents the time saved for different numbers of clusters, each of them obtained with respect to the distribution with one cluster less.

According to Figure 6, in general, each time a new station is added, the expected time saved is less significant than the previous one. Given that the province of Valencia currently has 26 forest fire stations, which lies in the interval [25,30], it can be affirmed that increasing the number of stations does not imply a great benefit. Therefore, it is believed that the research should focus on finding an optimized distribution for a number of stations between 20 and 26. Analyzing this interval, two important jumps can be identified. There are gains of 19${}^{\prime }$${}^{\prime }$ and 23${}^{\prime }$${}^{\prime }$ when adding the twenty-third station in $t_{avg}$ and $t_{w}$, respectively. Those jumps are only comparable to the addition of the twenty-fifth station—17${}^{\prime }$${}^{\prime }$ and 18${}^{\prime }$${}^{\prime }$ faster than the 24-station model. Thus, according to this reasoning, it was decided that the number of stations should be 23 or 25.

### 4.2. Optimized Location of Forest Fire Stations

**Figure 7:**
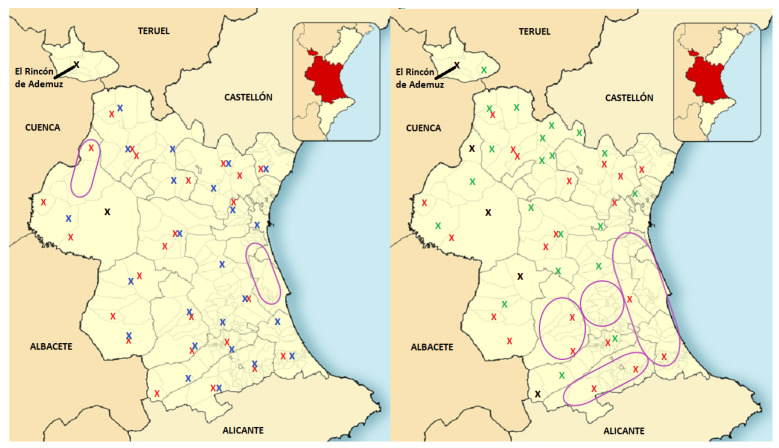
In the left image, the real stations (red Xs) are compared with those calculated by *k*-means (blue Xs), and in the right image, the same comparison is made with the results of running DBSCAN (green Xs).

Given that the current disposition of forest fire stations in the province of Valencia includes 26 units, the resulting distribution after executing *k*-means with 26 clusters has been chosen for an initial comparison, although it is believed that a model containing 23 or 25 clusters could provide practically the same results. For the sake of completeness, and to further show that the DBSCAN algorithm does not provide a suitable station relocation setup, its resulting distribution with 26 clusters has been also analyzed in this section. In order to provide a visual comparison between those two models and between each model and the current fire station deployment, Figure 7 presents two maps of the province where current fire stations are marked using a red X. The map on the left includes blue Xs to represent the cluster centers obtained through *k*-means. Similarly, green Xs are used to present the theoretically optimized placements according to DBSCAN on the right. Black Xs are used to represent the overlap of a real station with an optimized one. Areas surrounded by a violet ovals are the most unprotected regions in cases of fire, as there would be no stations nearby after each redistribution. Note that the unprotected areas are particularly large in the distribution proposed by DBSCAN.

As it can be seen (especially on the left image of Figure 7), some optimized stations are located in positions that are very close to some existing ones. For this reason, several pairs of homologous stations could be established, because they are located, practically, in the same area. This reasoning will be thoroughly detailed in Section 4.3, where a comparison between the current fire stations and the optimized ones is included.

### 4.3. Optimized Forest Fire Station Planning

The relocation of fire stations according to the results obtained in the previous section may involve high deployment costs, which in turn makes it an impractical solution. This is why it was also decided to propose an alternative model where the current position of the stations is compared with those optimized locations obtained by running the *k*-means method with 26 clusters.

As commented above, in many cases the clustering results provide centroids in positions that are close to the real fire stations, so pairs of homologous stations can be determined. Table 2 depicts a comparison between the current fire stations’ locations and the optimized locations provided by the *k*-means algorithm. When the current stations and the new ones are close to each other (i.e., homologous), both were placed in the same row. Nevertheless, there are also some new stations that are very far from any current ones and vice versa. For these cases, pairs of homologous stations were determined randomly, since there is apparently no relationship between the real stations and the optimized ones.

Positions of the different fire stations in Table 2 are expressed in terms of the row and column indices in the matrix P. Given two matrix positions $a=(a_{r},a_{c})$ and $b=(b_{r},b_{c})$, the distance between both positions can be obtained through the Euclidean distance calculation:

(6) $$dist\left(a,b\right)=\sqrt{{\left(a_{c}-b_{c}\right)}^{2}+{\left(a_{r}-b_{r}\right)}^{2}}.$$

Note that the result returned is not the real distance between two points, since this calculation assumes that the distance between two horizontally or vertically adjacent cells is 1, but in real maps it will depend on the road distance (in km) between adjacent municipalities. A fair option to convert dist(a,b) into a realistic distance is to multiply it by approximately 2.74 km, which corresponds to the average distance between cell centers in the considered map.

The fourth column in Table 2 indicates a qualitative distance measurement between each pair of fire stations: the currently existing and the optimized ones (calculated by *k*-means). According to the differences between the real stations’ positions and the ones calculated by *k*-means, the resulting stations were classified in three levels: equal/almost equal (dist(a,b) ≤ 1), similar (dist(a,b) ≤ 3) and too different (dist(a,b) > 3).

**Figure 8:**
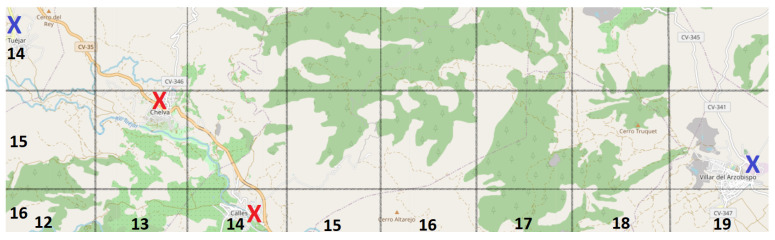
Small part of the province divided as the matrix P would do. The numbers at the bottom imply the column numbers and those on the left border the row numbers. The red Xs are placed where the current forest fire stations are, and the blue Xs represent the same stations in their optimized locations.

To illustrate the considered grouping levels, two examples are presented in Figure 8. Two existing fire stations (red Xs) and their corresponding optimized ones (blue Xs) appear. Visually, the red X in (15,13) and the blue X in (14,12) are diagonally adjacent, but they are not close enough to be classified as equal or almost equal, so they are classified as similar. On the other hand, there is apparently no relationship between the real station at (16,14) and the proposed one located at (15,19). By Equation (6), they are 5.1 far away (which in road distance means 13.97 km). Thus, they are labeled as too different due to the large distance between them.

As it can be observed in Table 2, there are eight stations of the current distribution that are located at very different coordinates when compared to the distribution proposed by *k*-means. These correspond to those labeled as too different and should be undoubtedly relocated.

Regarding the remaining fire stations, the best option would be certainly to also move all those ones that are not exactly in the same cell. Nevertheless, it is also true that the nearer the current and optimized stations, the shorter the gain in time in reaching a fire when moving the stations. In this way, we propose a sub-optimized fire station redistribution so that every fire station proposed to be located near an existing one will be moved to the position of such a real station in order to save relocation costs, at the expense of slightly reducing the overall performance.

Thus, 18 out of 26 forest fire stations could remain at their current places, so that only 8 stations are moved. Indeed, a model with 23 or 25 stations was found to be better than the one having 26 in Section 4.1. This way, at least one station could also be removed.

In order to remove three stations from the model obtained for 26 clusters in Table 2, we directly performed *k*-means with 23 clusters and removed the three stations that do no longer appear in the model. In this case, fire stations near centroids located at (24,29), (45,24) and (46,30) positions were removed by the clustering method, while the other fire stations were mainly placed at the same positions. As a result, the proposed final model is composed of 23 stations, and eight original stations should be moved, as they are classified as too different. These stations are the same as the ones labeled as too different in Table 2; the remaining ones could stay at their current locations.

### 4.4. Discussion of Results

In this section, the performance of the constrained model with 23 stations proposed at the end of the previous section (final proposal) is analyzed and compared with the previously presented results using *k*-means with full freedom to redistribute all stations. Table 3 compares the average and weighted times to reach a fire for the current distribution with 26 stations; the *k*-means distribution with either 23 or 26 stations; and the final proposal with 23 stations.

Currently, there are 26 stations, so if a logical rule was followed, one could expect that the fewer stations there are, the less protection there is. However, as already discussed, the use of *k*-means enhances the distribution of stations even when three stations less are deployed.

Regarding the proposed constrained distribution, it can be first observed that this model is not the fastest one in terms of $t_{avg}$. Nevertheless, its performance is still competitive, since its deviation with the *k*-means distribution with 23 stations is negligible (3${}^{\prime }$${}^{\prime }$). According to the $t_{w}$ results, the proposed distribution is considerably faster than the provided by *k*-means, with the advantage of avoiding the relocation of all the stations. These results have been obtained based on the parameters used to calculate distances, so the proposed distribution might be slightly different when varying those parameters.

**Figure 9:**
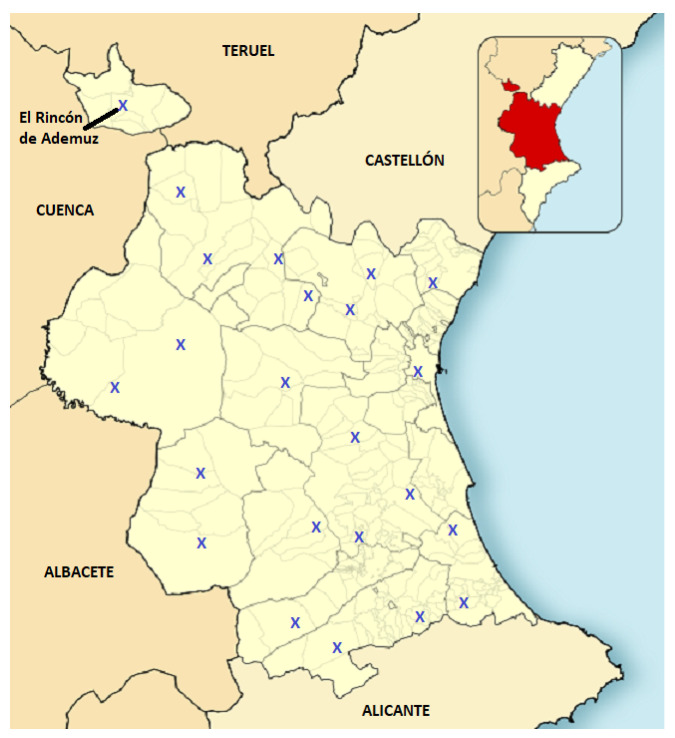
Distribution of forest fire stations in the province according to the proposed model.

The finally proposed distribution of fire stations is shown in Figure 9. We believe that the proposed relocation involves several economic benefits compared to unconstrained intelligent models. Indeed, the GVA might not be willing at first to make a high investment only to reach the forest fires a little bit faster. For this reason, we consider as an additional motivation the reduction of costs. To this end, a decrease in the number of fire stations from 26 to 23 is a first good measure to reduce the total maintenance cost. Furthermore, a great economic benefit could be obtained by selling the land where there used to be a station. In our view, these economic reasons could be a convincing argument to compensate for the necessary investment to move 8 stations.

Apart from that, it could be interesting to discuss about the scope that the project might have. Although the proposed model is tailored to the province of Valencia, the same process could be applied to any other region in order to obtain an optimized distribution of fire stations, as long as the necessary information about forest fires is available. A good road infrastructure allowing a rapid connection between different areas and fire stations is a fundamental part of this project. Therefore, in countries with an underdeveloped road network, it would probably be better to focus on optimizing the distribution of aerial means for fire fighting, where the aerial bases’ locations could be optimized following our proposed approach. Overall, we believe that the proposed method could be used on several areas of the planet, being of special interest for arid or fire-prone areas.

## 5. Conclusions

In this paper, we proposed an optimized redistribution of fire stations in the province of Valencia (Spain) aimed at minimizing the impact of forest fires. Using historical data about fires in the province, together with the location information about existing fire stations and municipalities, two different clustering techniques were applied to relocate the stations. Economic constraints have also been taken into account when making the final redistribution proposal.

The times to reach a fire obtained after relocating the fire stations using two clustering algorithms indicate that there are two opposite faces. On the one hand, we can conclude that the DBSCAN method is not useful for this problem, since it increases the times of the current distribution, even when the number of stations is greater than the number existing (26). The high density of data points in some regions, combined with the low density in others, worsens the whole performance. On the other hand, *k*-means clustering does fit well with the problem at hand, generally ensuring a faster arrival to the municipalities, according to the considered parameters, compared to the current distribution. Since it calculates cluster centers based on distances and not on the density, it is not affected by the latter problem.

However, as it was stated in the previous sections, the results of *k*-means should be modified to some extent, in order to obtain an economically viable proposal. We estimate that reducing the number of stations and not relocating current stations that are close to where they should be according to k-means would significantly reduce the project cost. Once the economical constraints have been considered in the final distribution, we believe that the proposal could help with the mitigation of forest fires through more rapid arrival at them. Furthermore, although the model is tailored to the province of Valencia, the same method could be used in almost any part of the world, thereby helping to combat wildfires globally.

## Figures and Tables

**Table 1:** Average and weighted times to reach a fire obtained with *k*-means and DBSCAN for different numbers of clusters between 20 and 30. Missing values for DBSCAN indicate that any optimized distribution was found with said number of clusters. Results with 26 clusters are highlighted in bold font to be compared with the existing distribution of stations (nowadays $t_{avg}$ = 12${}^{\prime }$37${}^{\prime }$${}^{\prime }$ and $t_{w}$ = 10${}^{\prime }$05${}^{\prime }$${}^{\prime }$).

Distribution	Time	Number of Fire Stations										
		20	21	22	23	24	25	26	27	28	29	30
***k*-means**	$t_{avg}$	11${}^{\prime }$22${}^{\prime }$${}^{\prime }$	11${}^{\prime }$10${}^{\prime }$${}^{\prime }$	11${}^{\prime }$02${}^{\prime }$${}^{\prime }$	10${}^{\prime }$43${}^{\prime }$${}^{\prime }$	10${}^{\prime }$42${}^{\prime }$${}^{\prime }$	10${}^{\prime }$25${}^{\prime }$${}^{\prime }$	**10${}^{\prime }$17${}^{\prime }$${}^{\prime }$**	10${}^{\prime }$11${}^{\prime }$${}^{\prime }$	9${}^{\prime }$55${}^{\prime }$${}^{\prime }$	9${}^{\prime }$52${}^{\prime }$${}^{\prime }$	9${}^{\prime }$47${}^{\prime }$${}^{\prime }$
	$t_{w}$	9${}^{\prime }$33${}^{\prime }$${}^{\prime }$	9${}^{\prime }$19${}^{\prime }$${}^{\prime }$	9${}^{\prime }$11${}^{\prime }$${}^{\prime }$	8${}^{\prime }$48${}^{\prime }$${}^{\prime }$	8${}^{\prime }$47${}^{\prime }$${}^{\prime }$	8${}^{\prime }$29${}^{\prime }$${}^{\prime }$	**8${}^{\prime }$18${}^{\prime }$${}^{\prime }$**	8${}^{\prime }$08${}^{\prime }$${}^{\prime }$	7${}^{\prime }$58${}^{\prime }$${}^{\prime }$	7${}^{\prime }$53${}^{\prime }$${}^{\prime }$	7${}^{\prime }$49${}^{\prime }$${}^{\prime }$
**DBSCAN**	$t_{avg}$	20${}^{\prime }$44${}^{\prime }$${}^{\prime }$	-	20${}^{\prime }$08${}^{\prime }$${}^{\prime }$	19${}^{\prime }$52${}^{\prime }$${}^{\prime }$	19${}^{\prime }$30${}^{\prime }$${}^{\prime }$	19${}^{\prime }$26${}^{\prime }$${}^{\prime }$	**19${}^{\prime }$21${}^{\prime }$${}^{\prime }$**	19${}^{\prime }$12${}^{\prime }$${}^{\prime }$	19${}^{\prime }$07${}^{\prime }$${}^{\prime }$	-	-
	$t_{w}$	19${}^{\prime }$13${}^{\prime }$${}^{\prime }$	-	18${}^{\prime }$07${}^{\prime }$${}^{\prime }$	18${}^{\prime }$02${}^{\prime }$${}^{\prime }$	17${}^{\prime }$40${}^{\prime }$${}^{\prime }$	17${}^{\prime }$38${}^{\prime }$${}^{\prime }$	**17${}^{\prime }$35${}^{\prime }$${}^{\prime }$**	17${}^{\prime }$34${}^{\prime }$${}^{\prime }$	17${}^{\prime }$33${}^{\prime }$${}^{\prime }$	-	-

**Table 2:** Comparison of current fire station coordinates in the province with the centroid positions obtained through *k*-means.

Fire Station Name	Position in P (Row,Column)		
	Current	*k*-Means	Comparison
La Font de la Figuera	(53,18)	(49,22)	Too different
Ontinyent	(52,26)	(52,27)	Almost equal
Castelló de Rugat	(49,33)	(48,32)	Similar
Ròtova	(47,37)	(47,39)	Similar
Enguera	(46,23)	(45,24)	Similar
Xàtiva	(45,29)	(46,30)	Similar
Ayora	(43,12)	(42,12)	Almost equal
Zarra	(41,11)	(41,28)	Too different
Navarrés	(41,23)	(40,22)	Similar
Alzira	(38,32)	(38,31)	Almost equal
Cortes de Pallás	(35,15)	(36,12)	Too different
Yátova	(29,19)	(41,36)	Too different
Los Isidros	(28,4)	(26,2)	Similar
Buñol	(28,20)	(28,21)	Almost equal
Requena	(25,10)	(25,10)	Equal
Villargordo del Cabriel	(24,0)	(33,27)	Too different
La Vallesa	(23,29)	(24,29)	Almost equal
Bétera	(21,30)	(21,26)	Too different
Pedralba	(20,22)	(20,19)	Similar
Gilet	(17,34)	(17,35)	Almost equal
Calles	(16,14)	(15,19)	Too different
Olocau	(16,28)	(16,29)	Almost equal
Sinarcas	(15,6)	(27,33)	Too different
Chelva	(15,13)	(14,12)	Similar
Titaguas	(10,10)	(9,12)	Similar
Ademuz	(2,4)	(2,4)	Equal

**Table 3:** Average and weighted times for the current distribution, the *k*-means distribution with two different numbers of stations and the proposal. The number of fire stations for each distribution is shown in parentheses.

Distribution	Current (26)	*k*-Means (26)	*k*-Means (23)	Proposal (23)
$t_{avg}$	12${}^{\prime }$37${}^{\prime }$${}^{\prime }$	10${}^{\prime }$17${}^{\prime }$${}^{\prime }$	10${}^{\prime }$43${}^{\prime }$${}^{\prime }$	10${}^{\prime }$46${}^{\prime }$${}^{\prime }$
$t_{w}$	10${}^{\prime }$05${}^{\prime }$${}^{\prime }$	8${}^{\prime }$18${}^{\prime }$${}^{\prime }$	8${}^{\prime }$48${}^{\prime }$${}^{\prime }$	**8${}^{\prime }$31${}^{\prime }$${}^{\prime }$**

## Data Availability

Data sources to reproduce the results are specified in the paper.
